# The Canada Journal of Dental Science

**Published:** 1868-07

**Authors:** 


### BIBLIOGRAPHICAL NOTICE.

*The Canada Journal of Dental Science.* Edited by W. George Beers, Montreal, and J. Stewart Scott, M. D., Toronto.

The first number of this Journal is before us and if its editors continue as they have commenced, it will certainly be worthy of support by the profession. This is the first attempt at establishing a Dental Journal in Canada, and we sincerely hope that it may meet with such encouragement as to enable its enterprising editors to persevere in their efforts "to improve and elevate the status of the profession in Canada.

The first number contains original articles from Drs. B. W. Day, C. S. Chittenden, H. Ross, and J. O'Donnell of Canada. Dr. W. H. Wait of England, and Dr. W. H. Atkinson of the United States.

